# Evaluation of Lipid Profiles of Children and Youth from Basic Health Units in Campinas, SP, Brazil: A Cross-Sectional Laboratory Study

**DOI:** 10.5935/abc.20190209

**Published:** 2020-01

**Authors:** Érica Ivana Lázaro Gomes, Vanessa Helena de Souza Zago, Eliana Cotta de Faria

**Affiliations:** 1 Universidade Estadual de Campinas - Patologia Clínica, Campinas, SP - Brazil; 2 Pontifícia Universidade Católica de Campinas (PUC - Campinas), Campinas, SP - Brazil

**Keywords:** Cardiovascular Diseases, Dyslipidemias, Hypercholesterolemia, Child, Young Adult, Unified Health System, Adolescent, Laboratory Test

## Abstract

**Background:**

Among dyslipidemias, hypercholesterolemia is considered the main risk factor for cardiovascular diseases in adults. In childhood and adolescence, elevated total cholesterol (TC) and low-density lipoprotein cholesterol (LDL-C) are positively associated with atherosclerosis markers, however, systematic screening for dyslipidemias in these groups is a controversial topic.

**Objective:**

To characterize the frequencies, types and severity of dyslipidemias in children and adolescents attended at the Basic Health Units managed by SUS in Campinas/SP.

**Methods:**

After an agreement with the Municipal Health Department of Campinas, consecutive results of serum lipid profiles (n = 312,650) of individuals of both sexes (n = 62,530) aged between 1 day old and 19 years were obtained, from 2008 to 2015. Age groups and dyslipidemias were classified according to recommendations in the literature. The statistical significance level adopted was the probability value (p) of 0.05 or less.

**Results:**

The observed frequencies of increased TC, triglycerides (TG), LDL-C and non-HDL-C (NHDL-C) were 33%, 40%, 29% and 13% respectively, and of reduced high-density lipoprotein cholesterol (HDL-C) the frequency was 39%. The frequencies, in general, were greater in females and in the southwest and south regions of the city, whose populations are more vulnerable from the socioeconomic point of view; on the other hand, in children and adolescents, the frequencies of TG and HDL-C prevailed, respectively.

**Conclusions:**

The high frequency and regionalization of dyslipidemias in children and adolescents indicate the need for specific actions in the handling and treatment of such diseases by the public health system of Campinas.

## Introduction

Cardiovascular diseases (CVD) represent one of the main causes of morbidity and mortality in Brazil and worldwide. According to the World Health Organization (WHO), in 2015 CVD accounted for 31% of deaths worldwide.^1^ In Brazil, 29% of deaths were due to CVD according to the Brazilian Society of Cardiology.^2^

Dyslipidemias play a well-established role in cardiovascular risk in adults, and so do hypertension, diabetes mellitus, early family history of coronary artery disease and smoking. Often these clinical situations are associated with comorbidities such as overweight, obesity, poor eating habits and physical inactivity, ^1,3^ with serious consequences for the individual and the public health system. ^4^

Children and adolescents account for 34% of the Brazilian population, an absolute contingent of 57,1 million people. ^5^ There is evidence that high levels of total cholesterol (TC) and low-density lipoprotein cholesterol (LDL-C) in childhood and adolescence are associated with atherosclerotic outcomes in young adults. In this context, Napoli et al. ^6^ demonstrated fatty streaks in the intrauterine life span, being more noticeable in pregnant women with hypercholesterolemia.

Unlike the Brazilian Dyslipidemia Directive (DBD) Update, universal lipid screening over two years of age as compared to that of children with risk factors^3^ was recommended, based on other studies, by Zachariah and Johnson^7^ for having a greater diagnostic sensitivity by 30% to 60%. In Brazil, there are few population studies involving dyslipidemias in childhood and adolescence. ^8^ In addition, the Study of Cardiovascular Risks in Adolescents (ERICA) stands out, with a national approach and covering a population of 80,000 young people between 12 and 17 years old. ^8^

A previous study in our laboratory characterized severe dyslipidemias in the juvenile population in a public hospital segment in Campinas. ^9^ However, there are still gaps regarding their characterization in regional terms. Thus, this study was designed to characterize the frequencies, types and severity of dyslipidemias in children and adolescents attended at the Basic Health Units (UBS) in Campinas, SP.

## Methods

This is a retrospective cross-sectional study based on monthly lipid profile databases, which were periodically sent to the Lipid Laboratory of the School of Medical Sciences of UNICAMP through an academic agreement with the Municipal Health Department of Campinas.

Between 2008 and 2015 312,650 results of serum laboratory tests were obtained from 62,530 individuals of both sexes, aged between one day and 19 years who visited UBS in the city of Campinas, SP, for medical outpatient care. The UBS are distributed over five regions or health districts of the city.

Only individuals with measured serum lipid profile were included in the study, with the following parameters: TC, triglycerides (TG), LDL-C, high-density lipoprotein cholesterol (HDL-C) and non-HDL-C (NHDL-C). These analyses were performed by enzymatic-colorimetric and/or direct homogenous methods for LDL, according to the quality control standards of the Brazilian Society of Clinical Pathology, including the blood collection stage in the UBS. NHDL-C was calculated. ^10^ A single chemical analyzer, Modular® Analytics Evo (Roche Diagnostics, Burgess Hill, West Sussex, UK), and reagents from Roche Diagnostics® (Mannheim, Germany) were used during the study period.

Dyslipidemias were classified biochemically in consonance with the cut-off values for age advocated by the current DBD^3^ as: isolated increases of LDL-C, TG, NHDL or reductions of HDL-C; mixed dyslipidemias, defined as lipid combinations of increased LDL-C and TG and/or increased LDL-C and reduced HDL-C and/or increased TG and reduced HDL-C.

We used the reference values of Kwiterovich PO^11^ for infants (children from one day to 23 months of age), once there are no DBD recommendations for this age group, except for TG in the 0-9 years of age group. For NHDL-C in all age groups, also absent in DBD recommendations, the desirable and undesirable values (<123 and ≥144) of Kwiterovich PO were used as well.

In order to determine the groups of children (2-11 years of age) and adolescents (12 to 19 years of age) we followed the Brazilian Child and Adolescent Statute definitions (Law n°. 8.069/90 updated with Law no. 12.010 of 2009), with adaptation of the upper limit for adolescents because of DBD, and the recommendations therein, between two and 19 years of age. ^3^ The cut-off values (mg/dL) used as desirable and undesirable are shown in Table 1.

**Table 1:** Reference values for lipids and lipoproteins in children and adolescents

Lipidic variables	Values (mg/dL)		
	Desirable	Undesirable (1 day to 23 months)	Undesirable (2 to 19 years)
TC	< 170	≥ 200	≥ 170
LDL-C	< 110	≥ 130	≥ 110
NHDL-C	< 123	≥ 144*	-
**TG**			
0-9 years	< 75	≥ 100	≥ 75**
10-19 years	< 90	≥ 130	≥ 90
HDL-C	> 45	< 35	≤ 45

Reference values for 1 day to 23 months old:

* NHDL-C ≥ 144 1 day to 19 years old

** TG ≥ 75 according to current DBD. TC: total cholesterol; LDL-C: low-density lipoprotein cholesterol; HDL-C: high-density lipoprotein cholesterol; NHDL-C: non high-density lipoprotein cholesterol; TG: triglycerides.

Lipid profiles were also evaluated for LDL-C ≥ 190 mg/dL, without concomitant hypertriglyceridemia, for laboratory characterization of possible cases of Familial Hypercholesterolemia (FH). ^10^

The city of Campinas is a Sao Paulo inland city that has approximately 1.1 million residents, ^12^ with 63 UBS distributed over five health districts: East (E), Northwest (NW), North (N), Southwest (SW) and South (S). ^13^

### Statistical analysis

The variables, either continuous or categorical, were analyzed using descriptive and comparative tests in software SPSS 24.0 (SPSS Inc., USA) and SAS 9.4 (Inc, Cary, NC, USA). Since there was only one lipid profile available for some individuals and for others several were available during the study, only a single and first lipid profile per year were used in the study period (2008-2015).

Tests were performed to verify the normality of data distribution (Kolmogorov-Smirnov). The groups were then compared by the Mann-Whitney and Kruskal-Wallis tests with Bonferroni post-test, with data presented as median and interquartile ranges for the continuous variables, and chi-square (X^2^) test with the post-test for multiple comparisons in contingency tables based on permutations for categorical variables. Values of p < 0.05 were considered significant.

## Results

**Figure 1:**
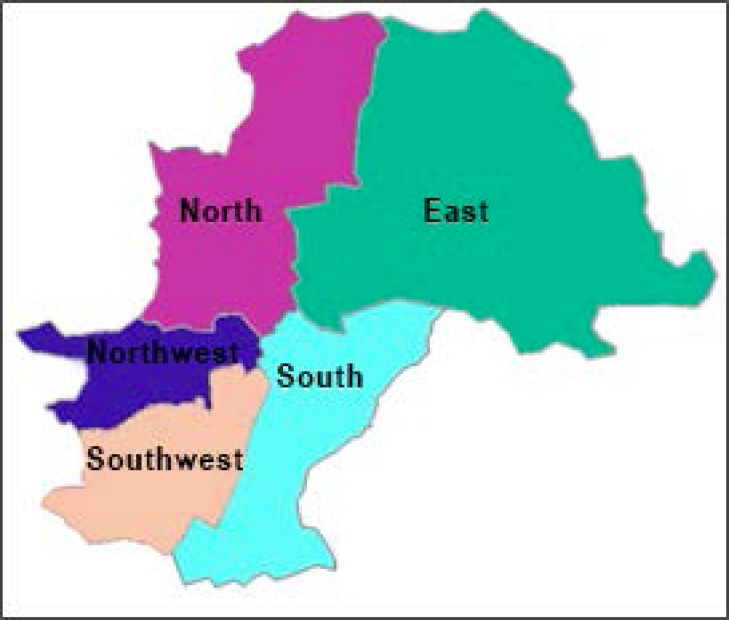
Corresponding area to the health districts in the map of the city of Campinas.

Source: Campinas Health Department.^13^

Figure 1 shows the distribution of the five health districts of Campinas with the respective numbers observed for test results, of individuals evaluated and their percentage frequencies: East: 41,075 and 8,215 (13%); Northwest: 61,900 and 12,380 (20%); North: 52,975 and 10,595 (17%); Southwest: 79,305 and 15,861 (25%) and South: 77,395 and 15,479 (25%). Half of the tests came from the southwest and south regions.

Table 2 summarizes the demographic characteristics of subjects, including the origin of clinical laboratory calls.

**Table 2:** Demographic characteristics of all subjects and by sex and age

Features	Number of individuals	Total frequencies total (%)
Total	62,530	100
**Sex**		
F	34,932	56
M	27,598	44
**Age**		
Infants	660	1
F	399	0.6
M	261	0.4
Children	25,501	41
F	13,219	21
M	12,282	20
Adolescents	36,369	58
F	21,314	34
M	15,055	24

N: number; F: female; M: male.

The results of the descriptive and comparative analyses are shown in Tables 3 and 4.

**Tabela 3:** Lipid profiles: medians and interquartile ranges for all subjects and stratified by sex and age

Groups	Lipids (mg/dL)	Total	Female	Male	p*	p†
All	TC	156 (137-178)	158 (139-179)†	154 (135-176)		0.000
	TG	76 (57-103)	78 (59-105)†	73 (54-100)		0.000
	LDL-C	95 (78-114)	96 (79-114)†	93 (77-112)		0.000
	HDL-C	49 (41-57)	49 ( 42-57)†	48 (41-57)		0.000
	NHDL-C	106 (88-127)	107 (89-128)†	104 (86-125)		0.000
Infants (I)	TC	172 (151-202)*	177 (153-206)†	167 (147-193)	0.000	0.019
	TG	91 (67-131)*	94 (68-132)	87 (64-119)	0.000	0.068
	LDL-C	108 (88-132)*	110 (88-134)	105 (88-128)	0.000	0.138
	HDL-C	46 (39-56)	47 (39-58)	45 (38-53)		0.063
	NHDL-C	125 (101-150)*	128 (101-153)	119 (102-147)	0.000	0.108
Children (C)	TC	162 (143-182)	162 (143-182)	161 (143-182)		0.901
	TG	75 (56-103)	79 (60-109)†	71 (53-97)		0.000
	LDL-C	99 (83-118)	100 (84-118)†	99 (83-117)		0.013
	HDL-C	50 (42-58)*	49 (41-57)	51 (43-60)†	0.000	0.000
	NHDL-C	110 (93-130)	111 (94-131)†	109 (92-129)		0.000
Adolescents (A)	TC	153 (133-174)	155 (136-177)†	147 (128-168)		0.000
	TG	76 (57-102)	76 (58-103)†	74 (56-102)		0.000
	LDL-C	91 (75-110)	93 (77-111)†	88 (72-107)		0.000
	HDL-C	48 (41-56)	49 (42-58)†	46 (39-54)		0.000
	NHDL-C	102 (84-123)	105 (86-125)†	99 (82-120)		0.000

F: female; M: male;

(†) Mann-Whitney, F vs. M; p < 0.05.

(*) Kruskal Wallis Post-hoc Bonferroni, I vs C vs A = TC, LDL-C, NHDL-C - I>C>A; TG - I>C=A; HDL-C - C>A>I, p < 0.05; Continuous variables appear as medians and interquartile ranges. TC: total cholesterol; TG: triglycerides; LDL-C: low-density lipoprotein cholesterol; HDL-C: high-density lipoprotein cholesterol; NHDL-C: non high-density lipoprotein cholesterol.

**Table 4:** Lipid profiles: medians and interquartile ranges for all subjects and by regions of campinas

Lipids (mg/dL)	East (E)	Northwest (NO)	North (N)	Southwest (SO)	South (S)	p
TC	158 (139-179)*	155 (136-177)	157 (138-178)	156 (136-177)	156 (137-177)	0,000
TG	77 (58-105)*	74 (56-101)	76 (57-103)	77 (58-105)*	75 (56-103)	0,000
LDL-C	96 (79-115)*	94 (77-113)	95 (79-114)	94 (78-113)	95 (78-113)	0,000
HDL-C	49 (42-58)	49 (41-57)	49 (42-57)	48 (41-56)*	49 (41-57)	0,000
NHDL-C	107 (89-128)*	104 (86-125)	106 (88-127)	106 (88-127)	105 (88-126)	0,000

(*) Kruskal-Wallis Post-hoc Bonferroni, TC: E > others; N > NW/SW; TG: E/N/SW>NW; E/SW>S; LDL-C: E>NW/SW/S; N>NW; HDL-C: E>S; SW < others; NHDL-C: E>S; NW<others; p < 0.05. Continuous variables appear as medians and interquartile ranges. TC: total cholesterol; TG: triglycerides; LDL-C: low-density lipoprotein cholesterol; HDL-C: high-density lipoprotein cholesterol; NHDL-C: non high-density lipoprotein cholesterol.

Table 3 shows that in all the age groups, TC, TG, LDL-C and NHDL-C were higher for infants. TG was similar in children and adolescents, and the median of HDL-C levels was higher in children than in the others. In relation to sex, the female group had higher values.

In the comparison by age group, the results also showed significant differences for the parameters evaluated in both sexes.

Table 4 clearly shows that there were higher results for TC in the eastern region than in the other regions. LDL-C levels were higher in the eastern region than in the northwest, southwest and south; NHDL-C values were also higher in this region than in the south and lower in the northwest region than in the others.

In the eastern, north, and southwestern regions, triglyceridemia was higher than in the northwestern region, and in the eastern and southwestern regions, it was higher than in the south. HDL-C in the southwestern region was lower than in the other ones and higher in the eastern region than in the south.

Table 5 shows the frequencies of dyslipidemia and their ratios by sex.

**Table 5:** Frequencies of isolated and mixed dyslipidemias in all subjects and by sex

Dyslipidemias	N	All (%)	N	F (%)	N	M (%)	Frequency ratios (%) (F/M)	p
Isolated dyslipidemias	41,689	67	23,572	38^*^	18,117	29	1.3	0.000
TC↑	20,759	33	12,213	19^*^	8,546	14	1.4	0.000
TG↑	24,703	40	14,527	23^*^	10,176	16	1.4	0.000
LDL-C↑	18,299	29	10,594	17^*^	7,705	12	1.4	0.000
HDL-C↓	24,210	39	13,120	21^*^	11,090	18	1.2	0.000
NHDL-C↑	7,847	13	4,665	8^*^	3,182	5	1.5	0.000
Mixed dyslipidemias	20,205	32	11,682	19^*^	8,523	14	1.4	0.000
LDL-C↑and TG↑	10,903	17	6,485	10^*^	4,418	7	1.5	0.000
HDL-C↓ and TG ↑	12,978	21	7,270	12	5,708	9	1.3	0.692
HDL-C↓ and LDL-C↑	6,960	11	3,901	6	3,059	5	1.3	0.742

F: female; M: male; Chi-square test (X^2^); F vs M; p < 0.05. TC: total cholesterol; TG: triglycerides; LDL-C: low-density lipoprotein cholesterol; HDL-C: high-density lipoprotein cholesterol; NHDL-C: non high-density lipoprotein cholesterol.

The most frequent dyslipidemias were isolated increases in TG and reduction of HDL-C. By sex, dyslipidemia frequencies were higher in females.

**Figure 2:**
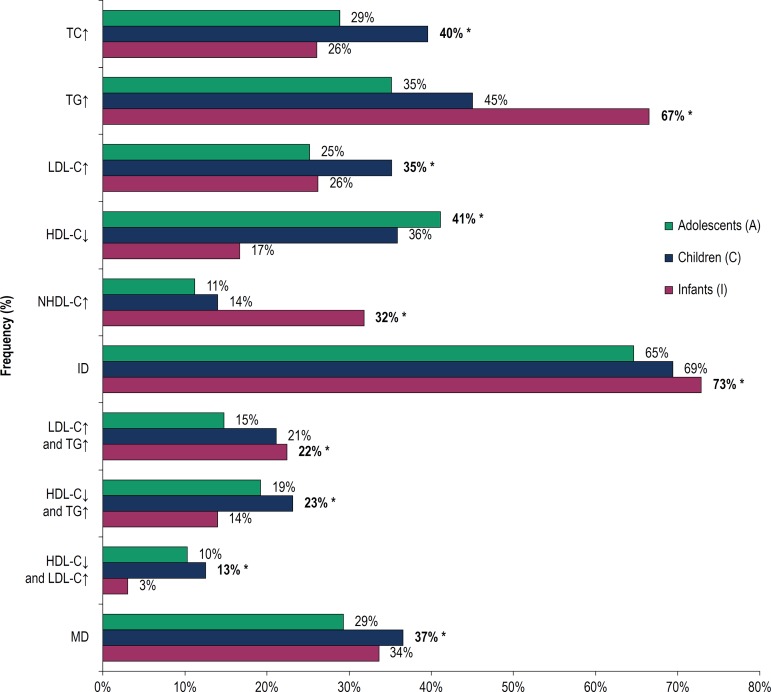
Frequencies of isolated and mixed dyslipidemias by age; ID: Isolated dyslipidemias; MD: Mixed dyslipidemias; *Chi-Square test (X^2^); I vs C vs. A; p <0.05; Post-test for multiple comparisons in contingency tables based on permutations: TC↑: LDL-C↑ - C > A = I; TG↑: NHDL-C↑ - I> C > A; HDL-C↓ A> C> I; HDL-C↓ and TG↑ and HDL-C↓ and LDL-C↑ - C> A> I; ID, LDL-C↑ and TG↑: MD-A <C = I; p <0.05. TC: total cholesterol; TG: triglycerides; LDL-C: low-density lipoprotein cholesterol; HDL-C: high-density lipoprotein cholesterol; NHDL-C: non high-density lipoprotein cholesterol.

Figure 2 shows the frequencies of dyslipidemia by age. Infants presented higher frequencies of increased TG and NHDL-C and isolated dyslipidemias, as well as a higher prevalence of the combination of increased LDL-C and TG.

As for children, higher levels of TC and LDL-C were observed, as well as the combination of increased TG and reduced HDL-C; there was also a higher frequency of at least one type of mixed dyslipidemia. On the other hand, adolescents showed a higher number of reduced HDL-C results.

**Figure 3:**
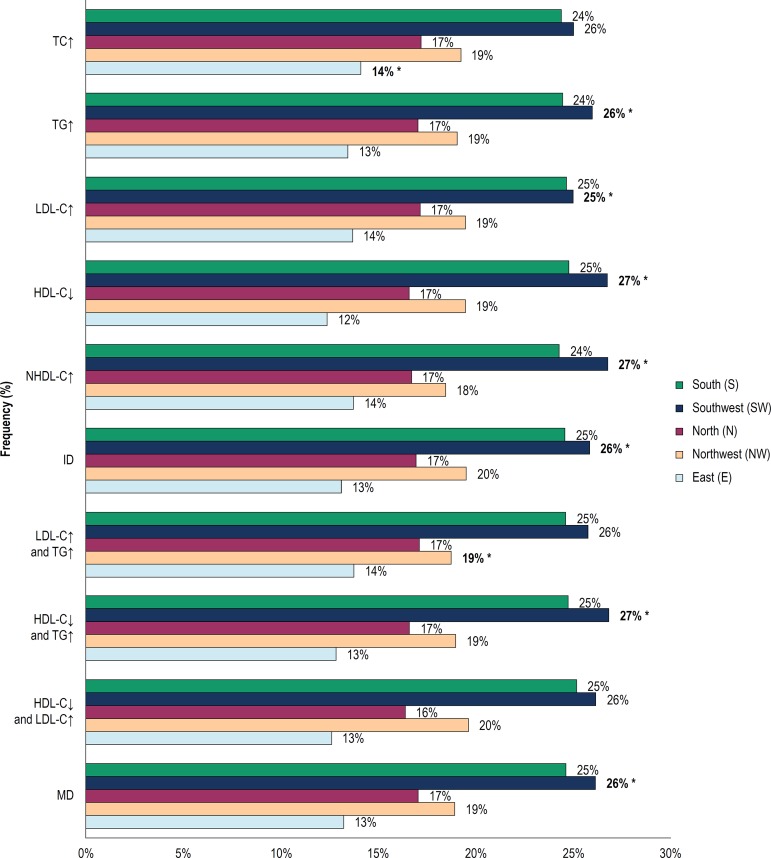
Frequencies of isolated and mixed dyslipidemias by regions of campinas; ID: Isolated dyslipidemias; MD: Mixed dyslipidemias; *Chi-Square test (X2); E vs NW vs N vs SW vs S; p <0.05; Post-test for multiple comparisons in contingency tables based on permutations: TC↑ = E <others; TG↑, NHDL-C↑ = SW> NW> E; LDL-C↑ = SW > E <NW; HDL-C↓ = SW> others, S> E; ID = NW <SW> S; LDL-C↑ and TG↑ = - NW> L; HDL-C↓ and TG↑ = SW> NW> N> E; MD = SW > NW, p < 0.05. TC: total cholesterol; TG: triglycerides; LDL-C: low-density lipoprotein cholesterol; HDL-C: high-density lipoprotein cholesterol; NHDL-C: non high-density lipoprotein cholesterol.

Figure 3 shows the frequencies of dyslipidemia by regions of Campinas.

Dyslipidemias were more frequent in the southwestern region of Campinas than in the other regions.

## Discussion

The development of atherosclerotic plaques is directly associated with an increase in NHDL-C lipoproteins, and evidence suggests that childhood cardiovascular risk factors such as dyslipidemia may contribute to atherosclerotic disease in childhood and adolescence, as well as in adulthood. ^14^

In this sense, the ongoing International Childhood Cardiovascular Cohort (i3C) Consortium^15^ aims to evaluate the association of the presence of risk factors in childhood with the outcomes of CVD morbidity and mortality in adults. Preliminary results showed that pediatric dyslipidemia predicts dyslipidemia^16^ and greater carotid intima-media thickness^17^ in adults. In addition, the presence of risk factors developed from the age of nine was predictive of subclinical atherosclerosis in adults. ^18^

In the present study, 67% of lipid profile results indicated the presence of at least one type of biochemically classified dyslipidemia. This percentage is greater than the one reported in other national studies: one of them, for example, carried out in the northeastern region of Brazil between 2011 and 2012, involving children and adolescents (6-18 years old), found the frequency of 62% for dyslipidemias with increases in TC and/or TG and/or LDL-C and/or reductions in HDL-C. ^19^ Another study in Londrina/PR with adolescents (11-16 years old) showed that 61% of the subjects had dyslipidemia (elevated TC and/or TG and/or LDL-C and/or reduced HDL-C). ^20^ Also, in 2007 a study of schoolchildren (10-14 years old) from Recife/PE described at least one type of dyslipidemia in 63.8% of the sample. ^21^

Other works performed in North and South American countries showed lower frequencies. In the United States^22^ between 2011 and 2014, in individuals aged 6 to 19 years, the reported frequency of dyslipidemia was 21% (increased TC and/or NHDL-C and/or reduced HDL-C).

In Santiago, Chile^23^ (2009-2011) in 2,900 individuals aged 10 to 14 years, the frequency of dyslipidemia was 32% (elevated TC and/or TG and/or LDL-C and/or reduced HDL-C). The higher prevalence in the present study may have been caused in part by the lower cut-off values ​​used by the national guideline^3^ when compared to the international guidelines. ^19,20^

In the analyses performed to evaluate the effect of sex, dyslipidemias were more frequent for all lipid parameters in females, and these results are consistent with national^24^ and international studies. ^25^ In fact, variations in serum lipoprotein levels are inherent to these individuals in the developmental stages and, consequently, to variations in sex hormones. ^26^

Some studies indicate that estrogens increase HDL-C in part due to their action in reducing hepatic lipase (HL) activity and increasing ATP-binding cassette transporter A1 receptors (ABCA1). ^27^ In addition, they decrease LDL-C^27^ levels by positively regulating LDL receptors, thus exerting a beneficial effect on the lipoprotein profile. ^28^

On the other hand, androgens increase HL activity, leading to an inverse effect: ^27^ HDL-C is reduced while LDL-C is increased. In contrast, Zhang et al. ^29^ indicated that testosterone may be associated with changes in SR-B1 receptor and HL activity, facilitating the selective uptake of HDL and playing an antiatherogenic role. ^29^

Comparisons by age groups revealed that infants presented higher levels of TG, NHDL-C and combination of LDL-C and TG as well as a high frequency of individual dyslipidemias; few studies report this data up to two years of age due to the difficulty of blood collection and metabolic instability in this phase of rapid growth before 24 months of life. ^30^ In addition, the high frequency of hypertriglyceridemia would occur through lactation and lack of food fasting. The current DBD defines the cut-off value of TG without fasting for the 0-9 years of age range as ≥ 85 mg/dL. Evaluating this interference, we applied that cut-off value, and the results showed a lower frequency, 56% instead of 67% (≥ 75 mg/dL, with fasting).

In this context, it is also worth noting that according to the national guidelines, ^10^ it is recommended to determine the lipid profile in children and adolescents when: i) grandparents, parents, siblings and first cousins ​​present dyslipidemia, mainly severe or with manifestation of premature atherosclerosis; ii) in the presence of clinical signs of dyslipidemia; in the presence of other cardiovascular risk factors; iii) with involvement of other pathologies, and iv) in the use of contraceptives, immunosuppressants and other drugs that may lead to dyslipidemia. ^31^ Therefore, it is expected that other factors, not collected here, would potentially justify these variations.

As for children, elevated TC and LDL-C were 40% and 35%, respectively. This increase in TC is close to that of a 2009 study with 217 individuals (84 obese), aged 2-9 years in Campina Grande/PB, ranging from 37% to 46%.^32^ Moreover, this result of TC in children is consistent with data from the National Health and Nutrition Examination Survey (NHANES) of individuals aged 4-19 years, where elevations of TC levels were observed in the 9-11 years age group, decreasing later along the pubertal development. ^33^

Ramos et al. (2011) ^32^ reported that the increase in LDL-C ranged from 14% to 14.8% in children (non-obese and obese), a finding that is lower than that of our study (35%). However, the cut-off value we used is lower than that of the referred population. In addition, similar results were observed in a study in Mexico with children from 2 to 10 years of age: 30% of subjects presented LDL-C ≥ 110 mg/dL. ^34^

As for adolescents, there was a high frequency of low HDL-C (41%), a value close to the one reported in the ERICA study, which was 47% among 38,069 schoolchildren, ^8^ results aligned with those of this study, even considering the different methodological approaches of the two studies.

Other national studies have shown important data. A study conducted in the Northeast, with individuals aged 6 to 18 years, showed a low HDL-C frequency of 41%,^19^ and another one carried out in Natal, RN, with students aged between 10 and 19 showed that 50% of the sample had this type of dyslipidemia. ^35^ Another study conducted in the metropolitan region of Guadalajara, Mexico with 132 individuals aged 5 to 15 years showed a lower prevalence (38.7%), but not very different from our findings. ^36^ The high frequency of reduced HDL-C in adolescents may be associated with young people's lifestyle, which involves inappropriate eating habits, overweight and physical inactivity. ^37^

It is worth mentioning that in this study, 349 individuals presented serum phenotype with LDL-C ≥ 190 mg/dL, that is in 0.56% (1:200) the results were suggestive for FH. ^10^

In relation to the regions of Campinas, the frequency of dyslipidemias was higher in the south and southwest than in the other regions. According to unpublished reports from the City Hall of Campinas, these regions have the highest number of records (25.7% and 27.6%, respectively) ^38^ in the *Cadastro Único*, a platform of the Federal Government that characterizes low-income families. In fact, according to Johansen et al., ^12^ they make up the so-called “poverty mountain range”, where there is a socioeconomic homogeneity not observed in the other regions. ^39^ Additionally, they are the ones that have a greater number of SUS users, accounting for 50% of the test results in this study.

Socioeconomic asymmetry can compromise the lifestyle of populations with direct repercussions on morbidity and mortality indicators. According to the WHO, currently three-quarters of deaths from cardiovascular disease are occurring in low and middle-income regions.^1^

The ERICA study showed significant increases in dyslipidemias in the north and northeast regions of the country (regions reportedly with the highest poverty indices in Brazil); ^40^ also, ERICA suggests that regional differences in dyslipidemias occur through the process of epidemiological transition, that is, regions may be at different stages. ^8^

This study evaluated the second most populous city in the state of São Paulo, located in the southeastern region of Brazil, where urban sprawl occurred without adequate planning and culminated in the expansion of occupation areas with the consequence for the population of inappropriate access to urban services. ^12^

### Limitations of the study

One of the limitations concerns the fact that the evaluated database is of secondary origin, with possible inaccuracies in the insertion of demographic data throughout all the processes. We are aware of the continued use of the quality control standards of the Brazilian Society of Clinical Pathology by the Municipal Laboratory of Campinas, which supplies the laboratory data.

In addition, since it was a cross-sectional study, it was not possible to evaluate the incidence of cases of dyslipidemia.

## Conclusion

This study shows the high frequency of atherogenic dyslipidemias in adolescents, children and infants attended in Campinas, with a greater distribution in the less favored socioeconomic regions, indicating the need for a regionalized focus during the development of public health programs for the prevention of early and adulthood CVD, including proper handling and treatment of dyslipidemias.
